# *Salmonella* Agona Harboring Genomic Island 1-A

**DOI:** 10.3201/eid1004.030417

**Published:** 2004-04

**Authors:** Benoît Doublet, Patrick Butaye, Hein Imberechts, Jean-Marc Collard, Elisabeth Chaslus-Dancla, Axel Cloeckaert

**Affiliations:** * Institut National de la Recherche Agronomique, Nouzilly, France; † Centre d’Etude et de Recherche Vétérinaires et Agrochimiques, Brussels, Belgium; ‡ Scientific Institute of Public Health, Brussels, Belgium

**Keywords:** *Salmonella*, Agona, SGI-A variant, human case, multidrug resistance gene cluster

**To the Editor**: Multidrug-resistant *Salmonella enterica* serovar Typhimurium definitive phage type 104 has emerged during the 1980s and 1990s as a world health problem because of its implications in animal and human disease (*1*–*3*). Epidemic serovar Typhimurium definitive phage type 104 isolates are commonly resistant to ampicillin (Ap), chloramphenicol (Cm)/florfenicol (Ff), streptomycin (Sm)/spectinomycin (Sp), sulfonamides (Su), and tetracyclines (Tc) (*1*,*3*). This multidrug-resistance phenotype is conferred by an antibiotic resistance gene cluster included in a 43kb genomic island named *Salmonella* genomic island 1 (*4*). *Salmonella* genomic island 1 has been recently characterized and located between the *thdF* and *int*2 genes of the chromosome. The *int*2 gene is part of a retron sequence found only in serovar Typhimurium. Downstream of the retron sequence is the *yidY* gene, which is also found in the chromosome of other *S. enterica* serovars. The antibiotic resistance gene cluster of approximately 13 kb is located at the 3’ end of *Salmonella* genomic island 1 (*4*). All resistance genes are clustered and are bracketed by two integron structures (*5*,*6*). The first integron carries the *aadA2* gene, which confers resistance to Sm and Sp. The second integron contains the β-lactamase gene *pse*-1, conferring resistance to Ap. Flanked by these two integron structures are the *floR* gene, which confers cross-resistance to Cm and Ff, and the tetracyclines-resistance genes *tetR* and *tet*(G) (*5*,*6*). Recently, *Salmonella* genomic island 1 has also been identified in other serovars of *S. enterica* namely Agona (*4*,*7*), Paratyphi B (*8*), and Albany (*9*), indicating the horizontal transfer potential of *Salmonella* genomic island 1. In these serovars, *Salmonella* genomic island 1 has the same chromosomal location as in serovar Typhimurium definitive phage type 104, except that they lack the retron sequence found downstream of *Salmonella* genomic island 1 (*4*,*8*,*9*). Moreover, six variant *Salmonella* genomic island 1 antibiotic resistance gene clusters (*Salmonella* genomic island 1-A to -F) have recently been reported for serovars Typhimurium DT104, Agona, and Albany to confer different multidrug resistance phenotypes (*9*,*10*). These clusters of genes were probably generated after chromosomal recombinational events or by antibiotic resistance gene cassette replacement in the integron structures. In particular, the *dfrA10* gene coding for trimethoprim (Tm) resistance was found downstream of the *pse-1* integron in a third unusual integron structure involving orf513 in the variant antibiotic resistance gene cluster called *Salmonella* genomic island 1-A (ApCmFfSmSpSuTcTm) (*10*).

Multidrug-resistant serovar Typhimurium definitive phage type 104 was disseminated globally with several outbreaks in humans and animals. At present, in contrast to the world health problem of multidrug-resistant serovar Typhimurium definitive phage type 104, human cases of infections with other *S. enterica* serovars harboring *Salmonella* genomic island 1 have not yet been reported. *Salmonella* genomic island 1-multidrug-resistant serovars Agona, Paratyphi B, and Albany were isolated from different animal species and countries (*7*–*9*). In this study, we analysed the first *Salmonella* genomic island 1 positive serovar Agona strain (02/01177) isolated from a human case in Belgium.

A Belgian patient, who had been infected by a multidrug-resistant serovar Agona strain was travelling to Turkey; subsequent to the multidrug-resistant serovar Agona strain, gastroenteritis developed. While in Turkey the patient sought medical care and was treated unsuccessfully with antimicrobial agents. Upon his return to Belgium, this serovar Agona strain was isolated from his stools, and he recovered after treatment with ciprofloxacin. The serovar Agona strain 02/01177 displayed the multidrug resistance profile ApCmFfSmSpSuTcTm, which suggested the possible occurrence of *Salmonella* genomic island 1-A (*10*). Moreover, the strain showed the same level of resistance to Ff as *Salmonella* genomic island 1 harboring *S.* *enterica* serovars (MIC of 64 µg/mL) (*7*–*9*).

To assess the presence of *Salmonella* genomic island 1 and its location in the chromosome, polymerase chain reactions (PCRs) were performed using primers corresponding to the left and the right (with or without retron) *Salmonella* genomic island 1 junctions to the chromosome as described previously (*4*,*8*–*10*). PCR results were positive for the left junction between the *thdF* gene of the chromosome and the *int* gene of *Salmonella* genomic island 1 (*4*). For the right junction, PCR results were positive between open reading frame (ORF) S044 of *Salmonella* genomic island 1 and *yidY* gene of the chromosome. Thus, these data indicate that this serovar Agona human isolate contains *Salmonella* genomic island 1 at the same chromosomal location as in other *Salmonella* genomic island 1 positive serovars but lacks the retron sequence found to date only in serovar Typhimurium strains (*4*,*8*,*9*).

PCR mapping of the typical antibiotic resistance genes and integrons associated with *Salmonella* genomic island 1 was realized as described previouly (*4*,*8*–*10*). PCR amplifications on genomic DNA extracted from serovar Agona strain 02/01177 yielded all specific fragments of the sizes expected from DNA of serovar Agona control strain 1169SA97 harboring *Salmonella* genomic island 1-A (data not shown) (*10*). These PCR mapping results indicated the presence of the typical *Salmonella* genomic island 1 resistance gene cluster with the insertion of the third unusual orf513 integron structure carrying *dfrA10* (*8*–*10*). These data are in accordance with the multidrug resistance phenotype of serovar Agona strain 02/01177 and indicate the presence of the variant antibiotic resistance gene cluster *Salmonella* genomic island 1-A (*10*).

Macrorestriction analysis by pulsed-field gel electrophoresis of DNA from serovar Agona strain 02/01177 cut by *Xba*I or *Bln*I, showed that this human isolate is indistinguishable by its *Xba*I or *Bln*I macrorestriction patterns from the other multidrug-resistant *Salmonella* genomic island 1-carrying serovar Agona strains isolated from poultry in Belgium (data not shown) (*7*). Thus, the human serovar Agona isolate appears clonally related to those from poultry.

To our knowledge, this is the first report describing a human infected by a serovar Agona strain harboring *Salmonella* genomic island 1-A. Moreover, it shows the first case where another *S. enterica* serovar harboring *Salmonella* genomic island 1 than the epidemic serovar Typhimurium definitive phage type 104 clone is implicated in human infection. The patient could probably have been infected before his travel to Turkey by a *Salmonella* genomic island 1-A carrying serovar Agona strain in Belgium where this type of strain is frequently isolated from poultry (Doublet et al., personal communication). This hypothesis is also supported by macrorestriction analysis, which showed that the strains from poultry and the human case-patient had identical *Xba*I and *Bln*I pulsed-field gel electrophoresis patterns and thus indicate that they are clonally related. Moreover, the patient was not in contact with poultry during his stay in Turkey and, to date, very little is known about the epidemiology of multidrug-resistant serovar Agona strains in this country. Further investigations on the epidemiology of multidrug-resistant serovar Agona strains harboring *Salmonella* genomic island 1 are warranted to avoid such strains becoming a worldwide epidemic, as was the case for multidrug-resistant serovar Typhimurium definitive phage type 104.

## References

[1] Cloeckaert A, Schwarz S. Molecular characterization, spread and evolution of multidrug resistance in *Salmonella enterica* serotype Typhimurium DT104. Vet Res. 2001;32:301–10. 10.1051/vetres:2001126 11432421

[2] Glynn MK, Bopp C, Dewitt W, Dabney P, Mokhtar M, Angulo FJ. Emergence of multidrug-resistant *Salmonella enterica* serotype Typhimurium DT104 infections in the United States. N Engl J Med. 1998;338:1333–8. 10.1056/NEJM199805073381901 9571252

[3] Ribot EM, Wierzba RK, Angulo FJ, Barrett TJ. *Salmonella enterica* serotype Typhimurium DT104 isolated from humans, United States, 1985, 1990, and 1995. Emerg Infect Dis. 2002;8:387–91. 10.3201/eid0804.010202 11971772 PMC2730249

[4] Boyd D, Peters GA, Cloeckaert A, Sidi Boumedine K, Chaslus-Dancla E, Imberechts H, Complete nucleotide sequence of a 43-kilobase genomic island associated with the multidrug resistance region of *Salmonella enterica* serovar Typhimurium DT104 and its identification in phage type DT120 and serovar Agona. J Bacteriol. 2001;183:5725–32. 10.1128/JB.183.19.5725-5732.2001 11544236 PMC95465

[5] Arcangioli MA, Leroy-Sétrin S, Martel JL, Chaslus-Dancla E. A new chloramphenicol and florfenicol resistance gene flanked by two integron structures in *Salmonella typhimurium* DT104. FEMS Microbiol Lett. 1999;174:327–32. 10.1111/j.1574-6968.1999.tb13586.x 10339826

[6] Briggs CE, Fratamico PM. Molecular characterization of an antibiotic resistance gene cluster of *Salmonella typhimurium* DT104. Antimicrob Agents Chemother. 1999;43:846–9. 10103189 10.1128/aac.43.4.846 PMC89215

[7] Cloeckaert A, Sidi Boumedine K, Flaujac G, Imberechts H, D’Hooghe I, Chaslus-Dancla E. Occurrence of a *Salmonella enterica* serovar Typhimurium DT104-like antibiotic resistance gene cluster including the *floR* gene in *S. enterica* serovar Agona. Antimicrob Agents Chemother. 2000;44:1359–61. 10.1128/AAC.44.5.1359-1361.2000 10770778 PMC89871

[8] Meunier D, Boyd D, Mulvey MR, Baucheron S, Mammina C, Nastasi A, *Salmonella enterica* serotype Typhimurium DT 104 antibiotic resistance genomic island I in serotype Paratyphi B. Emerg Infect Dis. 2002;8:430–3. 10.3201/eid0804.010213 11971780 PMC2730239

[9] Doublet B, Lailler R, Meunier D, Brisabois A, Boyd D, Mulvey MR, Variant *Salmonella* genomic island 1 antibiotic resistance gene cluster in *Salmonella enterica* serovar Albany. Emerg Infect Dis. 2003;9:585–91. 12737743 10.3201/eid0905.020609 PMC2972765

[10] Boyd D, Cloeckaert A, Chaslus-Dancla E, Mulvey MR. Characterization of variant *Salmonella* genomic island 1 multidrug resistance regions from serovars Typhimurium DT104 and Agona. Antimicrob Agents Chemother. 2002;46:1714–22. 10.1128/AAC.46.6.1714-1722.2002 12019080 PMC127246

